# Suppression of Crosstalk in Quantum Circuit Based on Instruction Exchange Rules and Duration

**DOI:** 10.3390/e25060855

**Published:** 2023-05-26

**Authors:** Zhijin Guan, Renjie Liu, Xueyun Cheng, Shiguang Feng, Pengcheng Zhu

**Affiliations:** 1 School of Information Technology, Nantong University, Nantong 226019, China; liu.rj@stmail.ntu.edu.cn (R.L.);; 2 School of Computer Science and Engineering, Sun Yat-sen University, Guangzhou 510006, China; 3 School of Information Engineering, Suqian College, Suqian 223800, China

**Keywords:** quantum circuit, crosstalk, command exchange rules, time stake, duration

## Abstract

Crosstalk is the primary source of noise in quantum computing equipment. The parallel execution of multiple instructions in quantum computation causes crosstalk, which causes coupling between signal lines and mutual inductance and capacitance between signal lines, destroying the quantum state and causing the program to fail to execute correctly. Overcoming crosstalk is a critical prerequisite for quantum error correction and large-scale fault-tolerant quantum computing. This paper provides an approach for suppressing crosstalk in quantum computers based on multiple instruction exchange rules and duration. Firstly, for the majority of the quantum gates that can be executed on quantum computing devices, a multiple instruction exchange rule is proposed. The multiple instruction exchange rule reorders quantum gates in quantum circuits and separates double quantum gates with high crosstalk on quantum circuits. Then, time stakes are inserted based on the duration of different quantum gates, and quantum gates with high crosstalk are carefully separated in the process of quantum circuit execution by quantum computing equipment to reduce the influence of crosstalk on circuit fidelity. Several benchmark experiments verify the proposed method’s effectiveness. In comparison to previous techniques, the proposed method improves fidelity by 15.97% on average.

## 1. Introduction

Quantum computing (QC) is a new computing mode that employs quantum information units to perform calculations based on quantum mechanical laws such as quantum entanglement and quantum superposition. Because of the superposition of quantum mechanics, quantum computing systems can solve some problems faster than traditional computers such as quantum image processing [1], cryptography [2], artificial intelligence [3], database search [4], and so on.

Quantum computing has advanced rapidly in recent years, with some public demonstrations of prototype quantum computing systems. Because hardware manufacturing technology is limited by many factors, including inaccurate quantum control and external interference [5], the noisy intermediate-scale quantum (NISQ) computing equipment will inevitably make mistakes in the execution of quantum circuits [6], limiting the execution ability of quantum computers [7,8]. As a result, developing new quantum algorithms and conducting quantum computing research on NISQ quantum computing equipment are critical for making the best use of scarce hardware resources and minimizing the noise impact of quantum algorithms on the equipment.

Crosstalk is a significant source of noise in NISQ quantum computing devices [9,10]. The driving signal focused on a specific qubit propagates to the adjacent qubit in NISQ hardware devices, resulting in crosstalk [11]. Previous research has shown that simultaneous execution of multiple gates causes significant crosstalk [9,10,11,12]. In various quantum computing devices, the error rate of quantum gates between every two qubits is 1–5% [13]. Crosstalk has been shown in studies to significantly increase the error rate and negatively impact the successful implementation of quantum circuits [9,10,12]. How to suppress crosstalk has emerged as a critical issue to address in order to improve the success rate of quantum circuits.

In the existing research, Murali et al. proposed crosstalk mitigation scheduling on noisy quantum computers, which uses the method of inserting barriers to mitigate crosstalk, but it will result in excessively long execution time of quantum circuits, which will inevitably lead to decoherence errors [9]. Lei Xie et al. proposed reordering instructions to reduce crosstalk in quantum computers, which greatly increased the parallelism of quantum circuit execution and reduced crosstalk [12].

However, there are a large number of executable quantum gates in NISQ quantum computing devices. Extending the instruction exchange rules will help reduce the noise impact caused by crosstalk even further. Meanwhile, different quantum gates in NISQ quantum computing devices have different durations [14]. Ignoring quantum gate duration may result in the simultaneous execution of multiple double quantum gates, which cannot effectively reduce crosstalk. In the implementation of NISQ quantum computing equipment, taking into account the difference in duration of different quantum gates can help reduce crosstalk caused by quantum circuits.

To address the aforementioned issues, this paper proposes multiple instruction ex-change rules for more executable quantum gates, which are then used to separate quantum gates with high crosstalk. Taking into account the duration of various quantum gates, a time stake insertion method is proposed in this process, by which the occupied state of qubits is updated to improve the parallelism of quantum circuits, and the influence of crosstalk on circuits is greatly reduced.

The rest of this paper is organized as follows: Section 2 introduces the fundamentals of quantum computing; Section 3 analyzes methods for reducing crosstalk in scheduling strategies; Section 4 and Section 5 describe the proposed multiple instruction exchange rules and the time stake insertion algorithm while taking into account different quantum gate durations; Section 6 discusses the experimental results; and Section 7 provides a summary of this paper.

## 2. Background of Quantum Circuit

### 2.1. Quantum Qubit and Quantum Gate

In a classical computer, information is stored in binary form, with two definite states, 0 and 1. In quantum computers, qubits, as the basic unit for storing information, also exist in two ground states: |0〉 and |1〉. Unlike the bits in classical quantum computers, qubits can be in a superposition state, which can generally be expressed as |φ〉=α|0〉+β|1〉, where ${\left|\alpha \right|}^{2}+{\left|\beta \right|}^{2}=1$, α and β are the probability amplitudes corresponding to the two ground states |0〉 and |1〉 [15].

**Figure 1:**
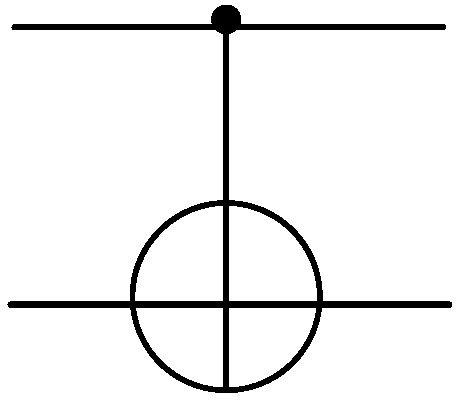
The CNOT gate.

Quantum gate is the basic operation performed on qubits in quantum computing. A single quantum gate acts on a single qubit, and a double quantum gate acts on two qubits. As shown in Figure 1. The CNOT gate flips the target qubit (represented by the ⊕ graph) if and only if the control qubit (represented by the black dot graph •) is in the |1〉 state. If the state of the control qubit is |1〉, the CNOT gate flips the state of the target qubit. If the state of the control qubit is |0〉, the state of the target qubit remains unchanged.

A quantum gate acting on qubits can be represented by a $2^{n}\times 2^{n}$ unitary matrix [16]. Quantum gates on NISQ quantum computing devices can be divided into single quantum gates and double quantum gates. In this paper, we consider the majority of the gates on NISQ quantum computing devices including single quantum gates such as X gate, Z gate, Y gate, H gate, T gate, T+ gate, S gate and S+ gate, as well as revolving gates such as RX(θ) gate, RY(θ) gate, RZ(θ) gate, $R_{z}^{+}(\theta )$ gate, and $R_{x}^{-}(\theta )$ gate. Double quantum gates include CNOT gate. Their corresponding symbols and unitary matrices are shown in Table 1.

### 2.2. Quantum Circuit and Unitary Matrix Calculation

**Figure 2:**
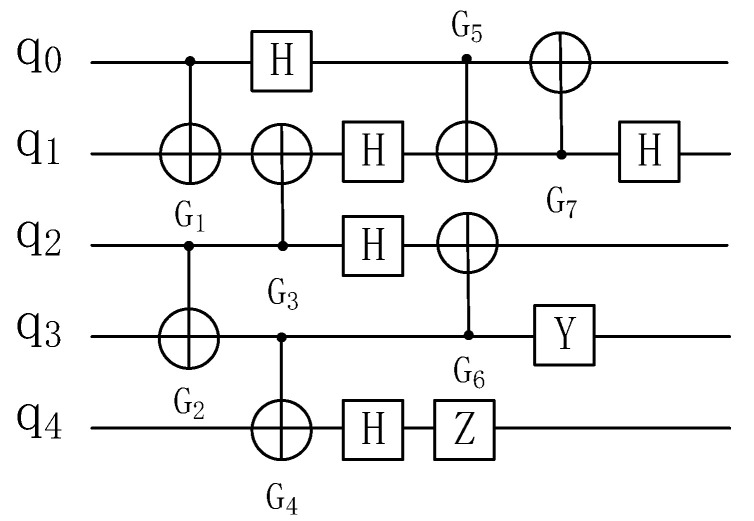
Example of a quantum circuit diagram.

A quantum circuit is a model used to describe a quantum algorithm in quantum computing [17]. A quantum circuit is made up of qubits and a series of quantum gates that act on these qubits. Figure 2 depicts a quantum circuit.

There are several functionally consistent quantum circuit representations in a quantum algorithm. The unitary matrix equivalence [18] can be used to determine whether two quantum circuits have equal functions. When two quantum circuits have the same functions, their unitary matrices are also the same. The symbolic representation of a unitary matrix in a quantum circuit is shown in Table 2, and the calculation rules are shown in Table 3.

### 2.3. Noise in NISQ Computing Equipment

Due to manufacturing technology limitations and other factors such as external interference, quantum computing equipment will inevitably produce noise. Table 4 displays the parameter information for NISQ computing equipment. The average error rate of single-qubit operations is less than 0.1%, and the error rate of double quantum gates between two qubits is between 2% and 8%, with an average of 3.8%.

## 3. Crosstalk Analysis in Scheduling

### 3.1. Hardware Causes of Crosstalk

In NISQ computing equipment, due to the defects of hardware manufacturing, the driving signal focused on a specific qubit will spread to the neighboring qubits, destroying their states and resulting in crosstalk. Existing methods for reducing crosstalk are classified as hardware strategies and scheduling strategies. Tunable couplers [19,20] and fixed-frequency qubit architectures [21,22] are two common hardware strategies. Although quantum computing devices are constantly slowing down crosstalk via hardware strategies [23,24], crosstalk still exists in actual quantum computing devices and has a significant impact on quantum circuit fidelity [23,25].

### 3.2. Reasons of Crosstalk in Scheduling

In this paper, we propose a crosstalk-mitigating scheduling strategy, which is a method for adjusting the quantum circuits that must be executed during the execution phase without changing their function. The difference between this strategy and the hardware strategy is that it focuses on quantum circuit optimization rather than quantum device hardware optimization.

To reduce the influence of crosstalk on the fidelity of quantum circuits through scheduling strategies, it is necessary to understand the causes of crosstalk during the execution of quantum circuits.

In order to explore the reason for crosstalk in scheduling strategies, this paper adopts the random benchmark test (RB) [26,27] to evaluate the quantum gate error rate. In the random benchmark test (RB), the error rate of a single quantum gate $G_{i}$, which is not affected by other quantum gates, is called the independent qubit error rate $E_{i}$. When $G_{i}$ and $G_{j}$ are measured simultaneously, the error rate of $G_{i}$ is called the conditional error rate $E_{i|j}$, while the error rate of $G_{j}$ is called the conditional error rate $E_{j|i}$. Its symbols and contents are shown in Table 5.

**Figure 3:**
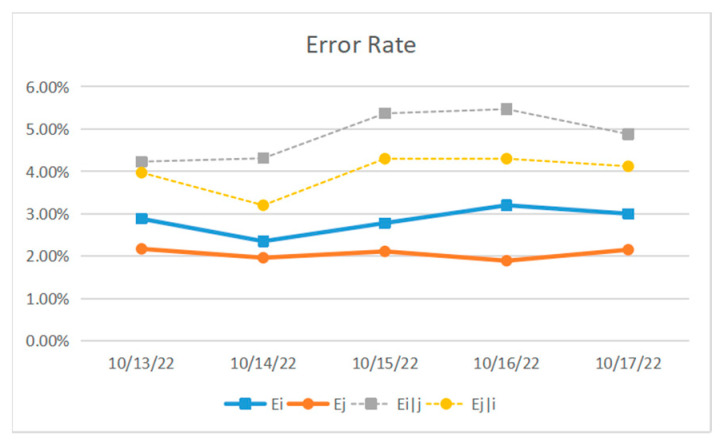
Daily independent qubit error rate and conditional qubit error rate on IBMQ 5 equipment.

Because the noise in NISQ computing equipment changes over time and space, the gate error rate (RB) evaluation will change. To better solve the crosstalk scheduling problem, IBMQ5 equipment was tested for independent qubit error rate and conditional qubit error rate for five consecutive days, and the relevant test result is shown in Figure 3.

Figure 3 shows that the independent error rates of $G_{i}$ and $G_{j}$ on IBMQ5 devices are around 2–3%, and the conditional error rates of $E_{i|j}$ and $E_{j|i}$ are nearly 4–6%. Figure 3 shows that all conditional qubit error rates are higher than independent qubit error rates, and simultaneous execution of gates CNOT will result in significant crosstalk, resulting in a more than doubled error rate.

**Definition** **1.** *When a single quantum gate or multiple quantum gates execute (simultaneously) in quantum computing devices, the noise generated by unwanted qubit interactions is called crosstalk.*

**Figure 4:**
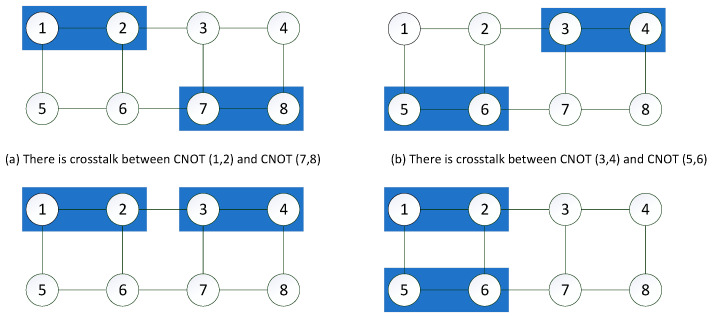
Crosstalk and high crosstalk.

Crosstalk presents two main risks. One is to decrease the accuracy of quantum gate execution, and the other is to increase the global impact of local quantum gates. In many leading architectures, crosstalk has been identified as the primary noise type. The interaction of quantum qubits causes crosstalk, especially when multiple quantum gates (instructions) are executed at the same time, as shown in Figure 4a,b. Due to the coupling effect between CNOT (1, 2) and CNOT (7, 8), CNOT (3, 4), and CNOT (5, 6). There is crosstalk between them.

When the crosstalk significantly affects the operational error rate of the quantum gate, it is called high crosstalk. In this paper, high crosstalk is referred to be the unintentional coupling between two adjacent parallel CNOT gates, as shown in Figure 4c,d.

### 3.3. Scheduling Strategy for Solving Crosstalk

This paper proposes a method of multiple instruction exchange rules and inserting time stakes to update the occupied state of qubits to solve the problem of high crosstalk. This method can separate double quantum gates with high crosstalk and reduce crosstalk from the standpoint of scheduling strategy.

## 4. Instruction Exchange Rules

Previous research has shown that separating quantum gates with high crosstalk and breaking the execution order between quantum gates can reduce the impact of crosstalk on the fidelity of quantum circuits, but previous research has only focused on a few quantum gates. This paper classifies the majority of the quantum gates in the executable quantum gate library on quantum computing devices and proposes the corresponding double-instruction exchange rules and multi-instruction exchange rules, which can separate most double quantum gates with high crosstalk at the logic quantum circuit level, effectively alleviating crosstalk.

### 4.1. Double Instruction Exchange Rules

#### 4.1.1. Partition of Gate Sets under Exchange Rules

On NISQ computing equipment, there are numerous executable quantum gates. We examine the majority of quantum gates used in quantum computing equipment including the following single quantum gates: X gate, Z gate, Y gate, T gate, $T^{+}$ gate, S gate and $S^{+}$ gate; revolving quantum gates: RX(θ) gate, RY(θ) gate, RZ(θ) gate, $R_{z}^{+}(\theta )$ gate, and $R_{x}^{-}(\theta )$ gate; double quantum gate: CNOT gate. As shown in Table 6, divide the above gates into gate sets.

#### 4.1.2. Double Exchange Rule

**Figure 5:**
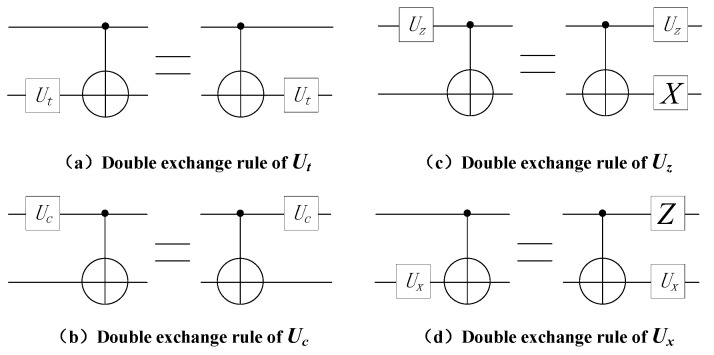
Double instruction exchange rule.

Previous research proposed a set of generalized exchange rules for some quantum gates; however, there are many executable quantum gates on quantum computing devices, and the generalized exchange rules only apply to a subset of them. As shown in Figure 5, this paper proposes a double instruction exchange rule for most executable quantum gates.

**Rule** **1.** *If the quantum $U_{t}$ gate is in the target position of the CNOT gate, the position of the two gates can be exchanged, and the quantum circuit before and after the exchange is equivalent. $U_{t}$ gate includes the X gate and the RX(θ) gate.*

**Proof** **of** **Rule** **1.** It is proved that if the unitary matrixes of quantum circuits are equal, they are functionally equivalent [18]. Therefore, we calculate the unitary matrix on both sides of the equation. For X gate in $U_{t}$, The unitary matrix on the left of the equation is (1). The unitary matrix on the right of the equation is (2), because (1) is equals (2). The equation holds. □

(1) $$(I_{n}⊗X)\times CT=\left[\begin{array}{cccc} 0 & 1 & 0 & 0\\ 1 & 0 & 0 & 0\\ 0 & 0 & 1 & 0\\ 0 & 0 & 0 & 1 \end{array}\right]$$

(2) $$CT\times (I_{n}⊗X)=\left[\begin{array}{cccc} 0 & 1 & 0 & 0\\ 1 & 0 & 0 & 0\\ 0 & 0 & 1 & 0\\ 0 & 0 & 0 & 1 \end{array}\right]$$

In the above formula, $I_{n}$ is the unit vector, CT is CNOT gate, and ⊗ is the tensor product.

**Rule** **2.** *If the quantum $U_{c}$ gate is in the control position of the CNOT gate, the position of the two gates can be exchanged, and the quantum circuit before and after the exchange is equivalent. $U_{c}$ includes Z gate, H gate, T gate, $T^{+}$ gate, S gate, $S^{+}$ gate and RZ(θ) gate.*

**Proof** **o** **Rule** **2.** For Z gate in $U_{t}$, the unitary matrix on the left side of the equation is the left side of (3). The unitary matrix on the left side of the equation is the left side of (3). Because their unitary matrices are equal, the equation holds. □

(3) $$\left(Z⊗I_{n})\times CT=CT\times (Z⊗I_{n}\right)$$

**Rule** **3.** *If the quantum $U_{z}$ gate is in the control position of the CNOT gate, after adding the quantum X gate to the target position of the CNOT gate, the positions of $U_{z}$ gate, X gate, and CNOT gate can be exchanged, and the quantum circuits before and after the exchange are equivalent. $U_{z}$ includes Y gate and $R_{z}^{+}(\theta )$ gate.*

**Proof** **of** **Rule** **3.** For $R_{z}^{+}(\theta )$ gate in $U_{t}$, the unitary matrix on the left side of the equation is the left side of (4), The unitary matrix on the left side of the equation is the left side of (4). Because their unitary matrices are equal, the equation holds. □

(4) $$\left(R_{z}^{+}(\theta )⊗I_{n})\times CT=CT\times (R_{z}^{+}(\theta )⊗X\right)$$

**Rule** **4.** *If the quantum $U_{x}$ gate is in the target position of the CNOT gate, after adding the quantum Z gate to the control position of the gate, the positions of the $U_{x}$ gate, Z gate, and the CNOT gate can be exchanged, and the quantum circuits before and after the exchange are equivalent. $U_{x}$ gate includes Y gate and $R_{x}^{-}(\theta )$ gate.*

**Proof** **of** **Rule** **4.** For $R_{x}^{-}(\theta )$ gate in $U_{t}$, the unitary matrix on the left side of the equation is the left side of (5). The unitary matrix on the left side of the equation is the left side of (5). Because their unitary matrices are equal, the equation holds. □

(5) $$\left(I_{n}⊗R_{x}^{-}(\theta ))\times CT=CT\times (Z⊗R_{x}^{-}(\theta )\right)$$

#### 4.1.3. Double Instruction Exchange Rules Reduce Crosstalk

**Figure 6:**
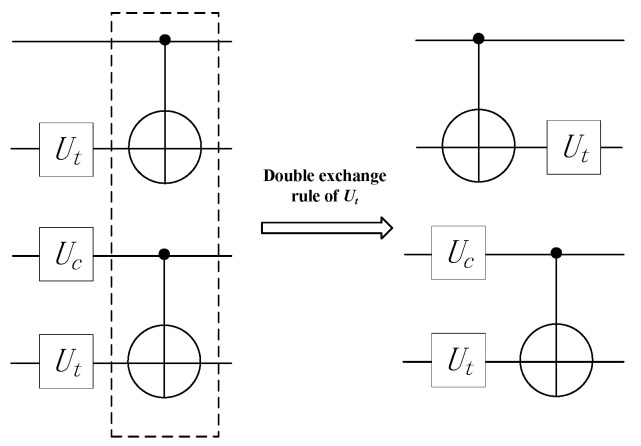
Double exchange rule reduces crosstalk.

The main source of crosstalk is the simultaneous execution of CNOT gates. As illustrated in Figure 6, instructions with high crosstalk can be separated through the double instruction exchange rule, reducing crosstalk.

### 4.2. Multiple Instruction Exchange Rules

**Figure 7:**
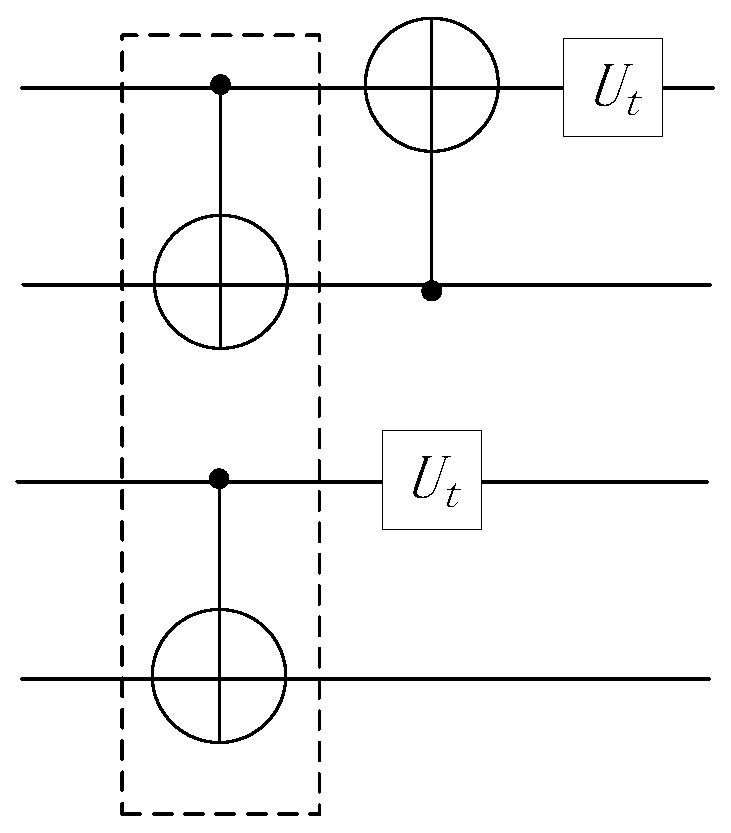
Crosstalk problem that cannot be solved by double exchange rules.

The double instruction exchange rule can alleviate crosstalk caused by a pair of CNOT gates executing simultaneously, but it cannot eliminate crosstalk caused by multiple CNOT gates running concurrently, as shown in Figure 7.

**Figure 8:**
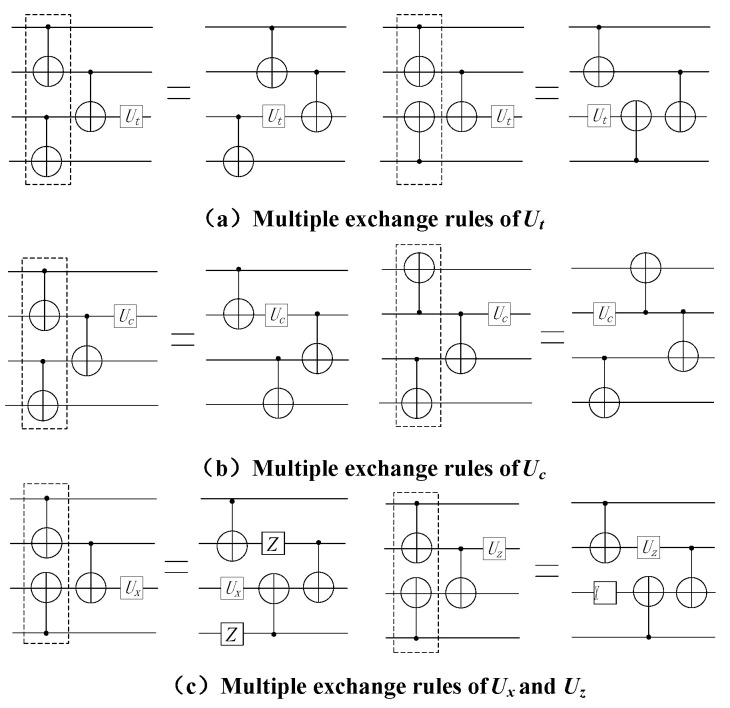
Multiple instruction exchange rules.

To solve the problem of crosstalk caused by multiple CNOT gates executing in parallel, which cannot be solved by double instruction exchange rules, this paper puts forward multiple instruction exchange rules to reduce crosstalk. On lines with high crosstalk caused by multiple CNOT gates executing in parallel, separating the CNOT gates with high crosstalk according to multiple instruction exchange rules can reduce the influence of crosstalk on quantum circuits. The multiple instruction exchange rules are shown in Figure 8.

**Rule** **5.** *Among the multiple CNOT, if the quantum gate $U_{t}$ is at the target position of the last CNOT or the quantum gate $U_{c}$ is at the control position of the last CNOT, the position of the last CNOT and $U_{t}$ /$U_{c}$ can be exchanged, and the CNOT with crosstalk in the previous layer can be separated to obtain an equivalent circuit with reduced crosstalk, as shown in Figure 8a,b.*

**Proof** **of** **Rule** **5.** It is proved that if the unitary matrixes of quantum circuits are equal, they are functionally equivalent. Therefore, we calculate the unitary matrix on both sides of the equation. For $U_{t}$ gate, the unitary matrix on the left side of the equation is the left side of (6). The unitary matrix on the left side of the equation is the left side of (6). Because their unitary matrices are equal, the equation holds. □

(6) $$\left(CT⊗CT)(I_{1}⊗CT⊗I_{1})(I_{2}⊗U_{t}⊗I_{1})=(I_{2}⊗CT)(CT⊗U_{t}⊗I_{1})(I_{1}⊗CT⊗I_{1}\right)$$

In the above formula, $I_{n}$ is the unit vector, CT is CNOT gate, and ⊗ is the tensor product.

**Rule** **6.** *Among the multiple CNOT, if the quantum gate $U_{x}$ is at the target position of the last CNOT or the quantum gate $U_{z}$ is at the control position of the last CNOT, the position of the last CNOT and $U_{x}$ /$U_{z}$ can be exchanged, and the CNOT with crosstalk in the previous layer can be separated to obtain an equivalent circuit with reduced crosstalk, as shown in Figure 8c.*

**Proof** **of** **Rule** **6.** For $U_{x}$ gate, the unitary matrix on the left side of the equation is the left side of (7). The unitary matrix on the left side of the equation is the left side of (7). Because their unitary matrices are equal, the equation holds. □

(7) $$\left(CT⊗U_{x}⊗Z)(I_{1}⊗Z⊗NCT)(I_{1}⊗CT⊗I_{1})=(CT⊗NCT)(I_{1}⊗CT⊗I_{1})(I_{2}⊗U_{x}⊗I_{1}\right)$$

## 5. An Update Algorithm of Qubit Occupation State Based on Inserting Time Stake

During the compilation of quantum programs, double instruction exchange rules and multiple instruction exchange rules can preliminarily separate quantum gates with high crosstalk and reduce the influence of crosstalk on quantum circuits.

However, the CNOT gates after preliminary separation can still be executed at the same time for some time in the actual quantum computing equipment execution process, and the high crosstalk cannot be completely reduced. To address the aforementioned issues, this paper proposes a method for updating the qubit occupation state by inserting time stakes, summarizes the durations of various quantum gates on NISQ computing equipment, inserts time stakes into quantum circuits based on the durations, and completely separates the simultaneous execution time with adjacent CNOT to reduce crosstalk.

### 5.1. Duration of Different Quantum Gates on Quantum Devices

Previous research on quantum circuit optimization assumed that different quantum gates were executed at the same time, but the execution times of different quantum gates in actual quantum computing devices were different [14]. As shown in Table 7, this paper summarizes the durations of various quantum gates on several NISQ computing devices.

**Figure 9:**
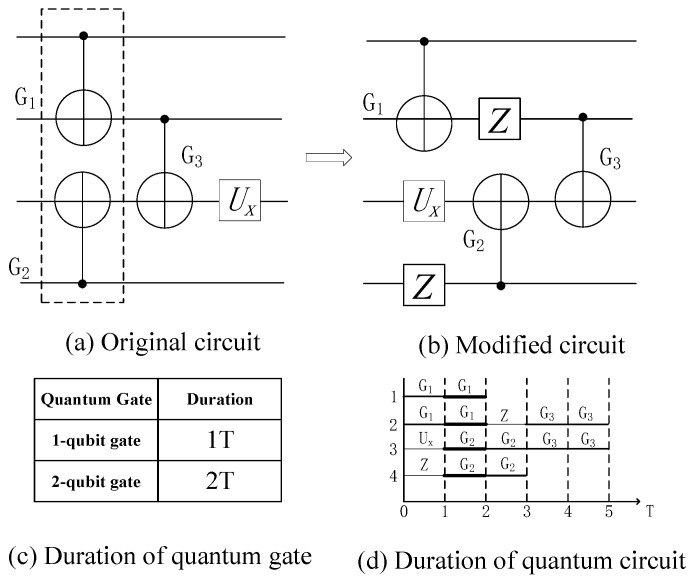
Influence of duration setting on crosstalk.

The data in Table 7 show that the duration of double quantum gates on different quantum computing devices is approximately twice that of single quantum gates. The duration of single quantum gates is set at one execution time cycle, and the duration of double quantum gates is set at two execution time cycles, according to the data in Table 8. Figure 9 depicts the effect of varying the duration on crosstalk.

In Figure 9a, the simultaneous execution of quantum gates $G_{1}$ and $G_{2}$ will result in significant crosstalk. Assuming that all quantum gates have a one-cycle duration, high crosstalk gates can be separated using multiple exchange rules; Figure 9b shows the equivalent quantum circuit obtained using multiple exchange rules, and Figure 9d shows the execution time of the quantum circuit on a real quantum computing device. The two quantum gates are executed simultaneously with one execution time cycle during the first and second time periods, and there is significant crosstalk.

### 5.2. Dependency GRAPH

The CNOT gates in the quantum circuit do not exist independently, and a double quantum gate $G_{i}$ occupying qubits $q_{i}$ and $q_{j}$ can only be executed after all previous double quantum gates $G_{j}$ have been executed, which is called $G_{i}$ depend on $G_{j}$ [28]. Traverse the whole quantum circuit, and construct a directed acyclic graph (DAG) according to this dependency, called relational dependency graph [29], to represent the dependency between two quantum gates in the quantum circuit. Its time complexity is O(g). The single quantum gate is not considered in the relation dependence graph because it can be executed on one qubit alone and will not have dependence on other qubits.

**Figure 10:**
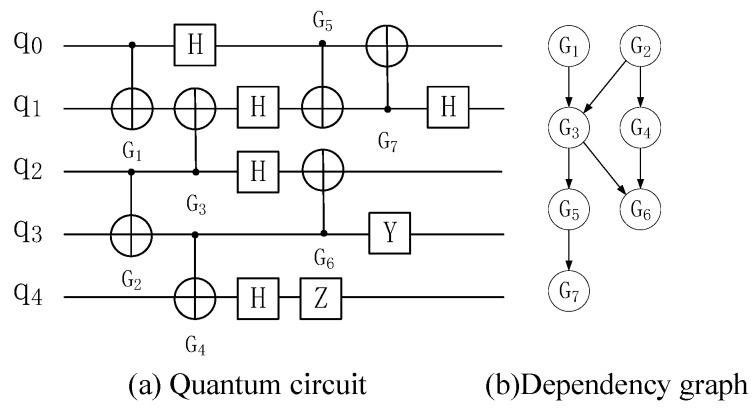
Dependency graph and layers.

An example is shown in Figure 10. Each node in the dependency graph represents a 2-qubit quantum gate $G_{i}$, and each directed edge represents the dependency of one 2-qubit quantum gate $G_{i}$ to another.

There are nodes with the degree of penetration of 0 in the dependency graph, which are recorded as $L_{1}$. Delete all nodes and directed edges in $L_{1}$ to obtain the second layer $L_{2}$. Traverse the whole quantum circuit in turn to obtain the layers of all quantum gates, as shown in Table 8.

### 5.3. Insert Time Stake

**Definition** **2.** *For a double quantum gate $G_{i}(q_{i},q_{i+1})$, the empty gate that occupies the adjacent qubits of the quantum gate for two execution periods and is used to update the occupied state of qubits is called a time stake, and its symbol is $G_{i.lock}(q_{i-1},q_{i+2})$. The time stake indicates that the qubit $q_{i-1}$ and $q_{i+2}$ cannot be occupied by other double quantum gates in two execution time cycles until the time stake is completed and the qubits are released from the occupied stake.*

A qubit cannot execute multiple quantum gates simultaneously in one time period; only one quantum gate can be executed at most [30]. If a quantum gate occupies a qubit for a certain period of time T, it is said that the qubit is in the occupied state for that period of time T. If other quantum gates want to apply to use the qubit, they need to wait for the qubit’s occupied state to be released.

The simultaneous execution of adjacent CNOT gates on a quantum circuit is the main cause of high crosstalk. Double exchange rules and multiple exchange rules can separate some CNOT gates with high crosstalk, but when they are executed on actual quantum computing devices, the adjacent CNOT gates still execute simultaneously in some time periods due to the different durations of different quantum gates.

For CNOT gate $G_{i}$ executed on qubits $q_{i}$ and $q_{i+1}$, it takes two units of time periods. If another CNOT gate $G_{j}$ is executed by adjacent qubits $q_{i-1}$ or $q_{i+2}$ in these two units of time periods, it will cause high crosstalk.

In order to solve the above problems, this paper sets a time stake $G_{i.lock}(q_{i-1},q_{i+2})$ for each double quantum gate $G_{i}(q_{i},q_{i+1})$ according to the layers of quantum gates in the relational dependency graph and separates the double quantum gates with high crosstalk by the occupation state of qubits. Traverse each double quantum gate in the Figure 10 quantum gate hierarchy and set a corresponding time post for it. A new layer of quantum gate, shown in Table 9, is obtained.

The hierarchical algorithm for obtaining quantum gates with time stakes by inserting time stakes into relational dependency graphs is as follows (Algorithm 1):
**Algorithm 1:** Insert time stakes to construct door setsInput: Dependency diagram corresponding to quantum circuit: DAG
Output: a hierarchical door set with time stakes. L
1       j← 1, LN=∅, L=∅
2       For each $G_{i}\in DAG$ do3          If $G_{i}.degress=0$ then4          $L_{j}$←$L_{j}.add(G_{i})$
5          DAG←$DAG.remove(G_{i}.edge)$
6          DAG←$DAG.remove(G_{i})$
7          LN←$LN.add(L_{j})$
8          j←1+19       For each $L_{j}\in LN$ do10          For each $G_{i}\in L_{j}$ do11
                  $L_{j}$←$L_{j}.add(G_{i.lock})$
12       L←$L.add(L_{j})$
13       Return L


### 5.4. Quantum Qubit State Update

The layered quantum gate after inserting the time stake completely separates the double quantum gate with high crosstalk, but the time stake is not an executable gate which cannot be directly executed on qubits and needs to be represented by the occupied state of qubits. For a qubit $q_{i}$, if it is occupied by two quantum gates $g_{1}$ and $g_{2}$ successively, it takes two units of time to execute the quantum gate. It is said that the qubit $q_{i}$ is occupied in these two units of time, and it is noted that $\delta T(q_{i})=2T$, and other double quantum gates cannot call the qubit in these two units of time.

According to the implementation of the quantum gate, the qubit state is constantly updated. As shown in Formula (8), before executing the double qubit gate $G_{k}$ and the corresponding time stake, the state of the qubits to be occupied must be updated.

(8) $$\delta T(q_{i})=\max\left\{TC+2T,\delta T(q_{i})+2T\right\}$$

TC is the current execution time of the quantum computing device, which represents a unit execution time cycle. Formula (8) indicates that the $q_{i}$ will not be released from the occupied state until the current execution time TC of the quantum computing device equals $\delta T(q_{i})$ and can be occupied by other double quantum gates.

After inserting the time stake, according to the layered quantum gate, all the double quantum gates and the time stakes are traversed in turn, and a new quantum gate is inserted according to the updated state of qubits so as to construct a new quantum circuit equivalent to the previous circuit. The quantum circuit effectively separates the CNOT gates with high crosstalk by using the time stakes insertion and the updated state of qubits. The quantum qubit occupancy state update algorithm is as follows (Algorithm 2):
**Algorithm 2:** Qubit occupation status updateInput: door set with time pile hierarchy L
Output: reconstructed quantum circuit LC
1          TC=∅, LC=∅2          For each $q_{i}\in q$ do3             If $\delta T(q_{i})$ ← 0 then 4          For each $L_{j}\in L$ do5             For each $G_{i}(q_{m},q_{n})\in L_{j}$ do6
                     LC←$LC.add(G_{i})$
7
                     $\delta T(q_{m})$←$\max\left\{TC+2T,\delta T(q_{i})+2T\right\}$
8
                     $\delta T(q_{n})$←$\max\left\{TC+2T,\delta T(q_{n})+2T\right\}$
9                 
TC=max{δT(q)}
10          Return LC


**Figure 11:**
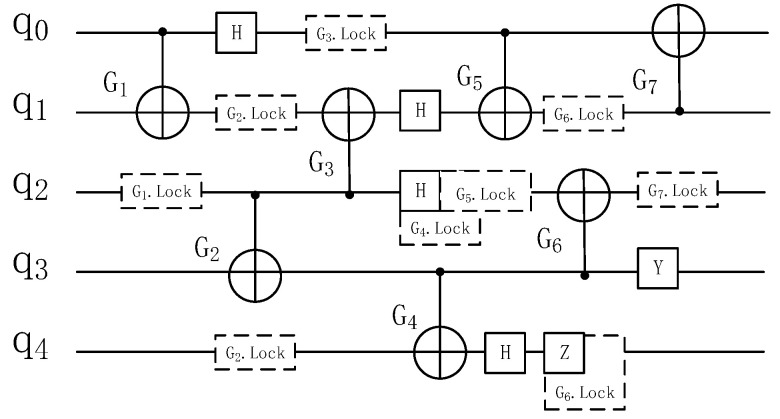
Quantum circuit after inserting time stake.

**Figure 12:**
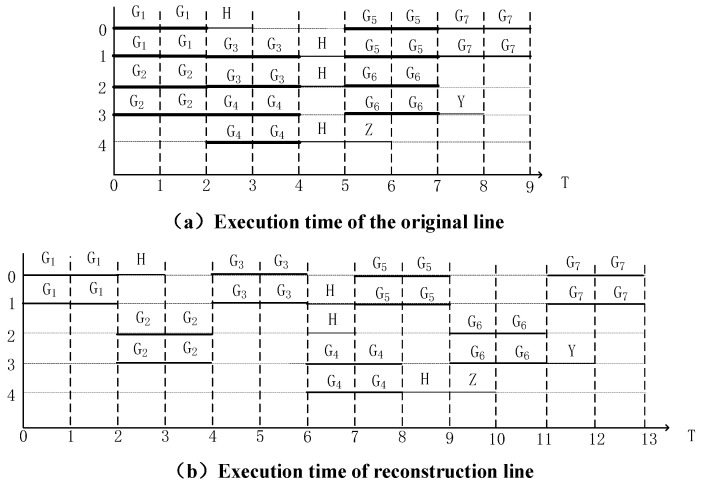
Crosstalk and execution time after inserting time stake.

Figure 11 shows the equivalent circuit diagram of the quantum circuit in Figure 10a after the qubit occupation state is updated. Figure 12 shows the total execution time of two quantum circuits on real quantum computing devices and the time period of crosstalk.

Figure 12 shows that three pairs of CNOT gates in the initial circuit will produce high crosstalk, which takes up six units of time cycles, and the execution time of the whole circuit is nine units of time cycles. The high crosstalk of the circuit after inserting the time stake takes up zero units of time cycles, and the execution time of the whole circuit is thirteen units of time cycles.

Previous studies have proved that the depth that can be increased by reducing a high crosstalk line is about ten units of time cycles [12]. Compared with the prior research [9], the method of inserting time stakes according to the duration of doors proposed in this paper separates most high crosstalk doors on the basis of a small increase in depth, effectively reducing the influence of crosstalk.

## 6. Experiment Result and Analysis

In this section, the crosstalk mitigation effect of the proposed method will be evaluated and analyzed.

### 6.1. Experiment Setup

The methods mentioned in this paper are all programmed in Python, in which we used the qiskit toolkit. The experimental environment CPU is an Intel (R) Core (TM) i7-8750H CPU @ 2.20 GHz, with 16GB of memory and the Windows S11 operating system. The benchmark circuit is selected from the RevLib benchmark data set [31] to carry out the exchange rules and circuit reconfiguration experiments.

In order to obtain a more accurate quantum circuit execution result, this paper uses the topological structure and parameter information of real quantum computing devices IBMQ Manila and Belem [32] to perform the experiment and uses IBMQ API [33,34] to instantly obtain the calibration data of real quantum computing devices, including quantum gate error rate and duration. The duration of all single quantum gates is set to one unit of time cycle, and the duration of double quantum gates is set to two units of time cycles.

### 6.2. Index

In this paper, the topology and parameter information of IBMQ Manila and Belem are used to evaluate the proposed algorithm. Using independent error rate and conditional error rate to simulate gate error. Each benchmark test is performed on IBMQ Manila and Belem, and 6000 experiments are performed on real quantum computing devices for each benchmark circuit. Take the number of successful experiments as an indicator to measure fidelity. This is a commonly used measure in previous experimental studies [12,35,36].

For example, a fidelity of 0.35 means that the number of expected results is 2100 among the results of 6000 experiments, accounting for 35% of the total number of experiments; the ideal fidelity is 1, which is the final result that quantum computing wants to achieve on real quantum computing devices in the future; and the quantum computing is completely correct.

### 6.3. Analysis of Fidelity Experimental Results

**Figure 13:**
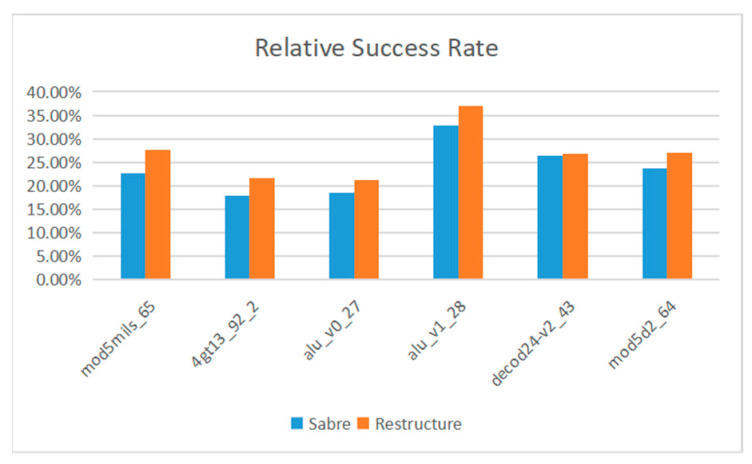
IBM Manila.

**Figure 14:**
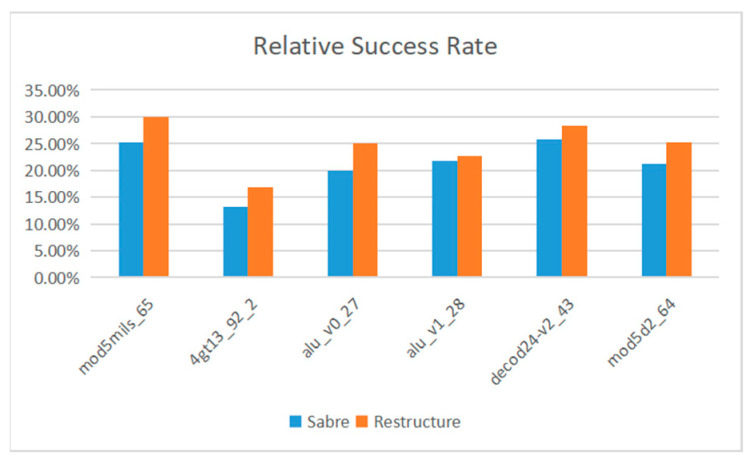
IBM Belem.

The Sabre [37] method proposed by G Li et al. is an advanced algorithm at present. We compare the fidelity of Sabre and the method proposed in this paper on Manila and Belem. Figure 13 and Figure 14 show the fidelity of the proposed method and Sabre in all benchmark tests.

(9) $$OR=\sum_{i=1}^{n}\frac{\mathrm{ED}-\mathrm{SA}}{TT}/\sum_{i=1}^{n}i$$

The formula for calculating the average optimization rate is shown in Formula (9), where OR is the average optimization rate, ED represents the number of successful quantum circuit experiments obtained by our proposed method, and SA represents the number of successful quantum circuit experiments obtained by using the Saber algorithm. TT represents the total number of tests.

On IBMQ Manila, the average optimization rate of the proposed method is 14.47%. On IBMQ Belem, the average optimization rate of the proposed method is 17.46%. The average optimization rate on the two devices is 15.97%.

### 6.4. Analysis of Experimental Results of Crosstalk Mitigation

**Figure 15:**
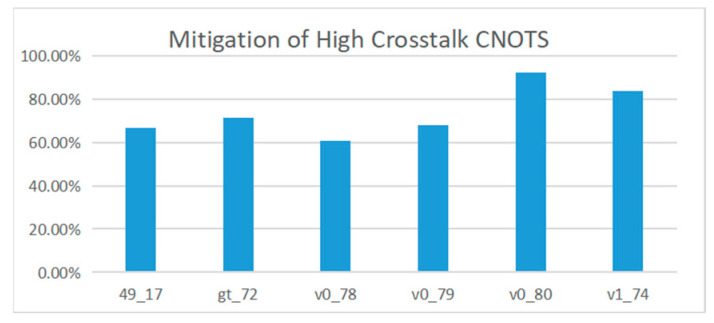
Reduction in high crosstalk in the quantum circuit.

Due to the defects in the hardware of quantum computing devices, all kinds of noise will be generated when the quantum computing devices execute, which makes the fidelity of quantum computing devices fail to reach the ideal state. High crosstalk is a major source of noise [12,23]. The method proposed in this paper can separate the double quantum gates with high crosstalk before the quantum computing equipment is executed and reduce crosstalk. Figure 15 shows crosstalk mitigation information for multiple reference circuits. The results show that the proposed method can effectively reduce the crosstalk in the line, and the average optimization rate reaches 79.78%.

## 7. Conclusions

Crosstalk is the primary source of noise in NISQ quantum computing equipment, and the simultaneous parallel execution of multiple double quantum gates is the primary cause of high crosstalk, which destroys quantum states on qubits, resulting in erroneous quantum circuit execution results. This paper proposes a method for updating the qubit occupation state using multiple exchange rules and inserting time stakes. Double quantum gates with high crosstalk are separated by multiple exchange rules and time stakes based on the duration of different quantum gates. Experiments show that the proposed method is very effective in reducing high crosstalk in quantum circuits and that, when compared to the prior art, the proposed method improves fidelity by 15.97% on average.

A quantum algorithm needs to go through several stages, from generation to actual operation. The process is as follows: conversion of quantum algorithms into logical quantum circuits; qubit mapping; quantum circuit routing; and quantum circuit scheduling. This study aims to mitigate crosstalk in logic quantum circuits and quantum circuit scheduling. In the future, we can also consider mitigating crosstalk in other processes to achieve better quantum circuit operation.

## Figures and Tables

**Table 1:** Quantum gate symbol and corresponding unitary matrix.

Symbol	Unitary Matrix	Symbol	Unitary Matrix
X	$\left[\begin{array}{cc} 0 & 1\\ 1 & 0 \end{array}\right]$	H	$\frac{1}{\sqrt{2}}\left[\begin{array}{cc} 1 & 1\\ 1 & -1 \end{array}\right]$
Z	$\left[\begin{array}{cc} 1 & 0\\ 0 & -1 \end{array}\right]$	RX(θ)	$\left[\begin{array}{cc} \cos(\theta /2) & -i\sin(\theta /2)\\ -i\sin(\theta /2) & \cos(\theta /2) \end{array}\right]$
Y	$\left[\begin{array}{cc} 0 & -i\\ i & 0 \end{array}\right]$	RY(θ)	$\left[\begin{array}{cc} \cos(\theta /2) & -\sin(\theta /2)\\ \sin(\theta /2) & \cos(\theta /2) \end{array}\right]$
S	$\left[\begin{array}{cc} 1 & 0\\ 0 & i \end{array}\right]$	RZ(θ)	$\left[\begin{array}{cc} e^{-i\theta /2} & 0\\ 0 & e^{i\theta /2} \end{array}\right]$
$S^{+}$	$\left[\begin{array}{cc} 1 & 0\\ 0 & -i \end{array}\right]$	$R_{z}^{+}(\theta )$	$\left[\begin{array}{cc} 0 & e^{i\theta /2}\\ e^{-i\theta /2} & 0 \end{array}\right]$
T	$\left[\begin{array}{cc} 1 & 0\\ 0 & e^{\frac{i\pi }{4}} \end{array}\right]$	$R_{x}^{-}(\theta )$	$\left[\begin{array}{cc} \cos(\theta /2) & -i\sin(\theta /2)\\ i\sin(\theta /2) & \cos(\theta /2) \end{array}\right]$
$T^{+}$	$\left[\begin{array}{cc} 1 & 0\\ 0 & e^{\frac{-i\pi }{4}} \end{array}\right]$	CNOT	$\left[\begin{array}{cccc} 1 & 0 & 0 & 0\\ 0 & 1 & 0 & 0\\ 0 & 0 & 0 & 1\\ 0 & 0 & 1 & 0 \end{array}\right]$

**Table 2:** Symbolic representation of unitary matrix.

Symbol	Represent Content
$U_{n}$	Unitary matrix of size $2^{n}\times 2^{n}$
$I_{n}$	Identity matrix of size $2^{n}\times 2^{n}$
$U_{B}$	Unitary matrix composed of control qubits and single quantum gates
$U_{D}$	Unitary matrix composed of qubits and single quantum gates
CT	Unitary matrix of a CNOT gate
NCT	The unitary matrix of the CNOT gate in which the control qubit and the target qubit swap qubits(inverted-CNOT)

**Table 3:** Calculation rules of unitary matrix.

Type	Pattern	Computing Formula
The quantum gate is below.	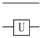	$U_{D}=I_{n}⊗U_{n}$
The quantum gate is above.	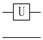	$U_{D}=U_{n}⊗I_{n}$
The control is above.	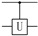	$U_{B}=|0\rangle \langle 0|⊗I_{n}+|1\rangle \langle 1|⊗U_{n}$
The control is below.	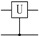	$U_{B}=I_{n}⊗|0\rangle \langle 0|+U_{n}⊗|1\rangle \langle 1|$

**Table 4:** Parameter information of superconducting computing equipment.

Fidelity	IBMQ5	IBMQ7	IBMQ16	IBMQ27	IBMQ65
Single qubit gate	99.9%	99.9%	99.9%	99.6%	98.9%
Double qubit gate	97.6%	96.8%	98%	92%	96.4%

**Table 5:** Symbol and content of qubit error rate.

Symbol	Represent Content
$E_{i}$,$E_{i}$	Independent qubit error rate unaffected by other quantum gates
$E_{i|j}$	The conditional qubit error rate of gate $G_{i}$ while gate $G_{j}$ is executed
$E_{j|i}$	The conditional qubit error rate of gate $G_{j}$ while gate $G_{i}$ is executed

**Table 6:** Partition of quantum gate set.

Symbol	Gate Set
$U_{c}$	{Z, H, T, $T^{+}$, S, $S^{+}$, RZ(θ)}
$U_{t}$	{X, RX(θ)}
$U_{x}$	{Y, $R_{x}^{-}(\theta )$}
$U_{z}$	{$R_{z}^{+}(\theta )$}

**Table 7:** Duration of different quantum gates on quantum computing devices.

Device Name	Single-Qubit Gate Duration	Double-Qubit Gate Duration
IBMQ5	116 ns	235–370 ns
IBMQ7	151 ns	284–640 ns
IBMQ16	113 ns	263–775 ns

**Table 8:** Quantum gate stratification in dependency graph.

Layers	Gate Set
*L*_1_	*G*_1_(*q*_0_, *q*_1_), *G*_2_(*q*_2_, *q*_3_)
*L*_2_	*G*_3_(*q*_2_, *q*_1_), *G*_4_(*q*_3_, *q*_4_)
*L*_3_	*G*_5_(*q*_0_, *q*_1_), *G*_6_(*q*_3_, *q*_2_)
*L*_4_	*G*_7_(*q*_1_, *q*_0_)

**Table 9:** Quantum gate layering inserted into time pile.

Layers	2-Qibit Gates and Time Stake Gate
*L*_1_	*G*_1_(*q*_0_, *q*_1_), *G*_1*.lock*_(*q*_2_), *G*_2_(*q*_2_, *q*_3_), *G*_2_(*q*_1_, *q*_4_)
*L*_2_	*G*_3_(*q*_2_, *q*_1_), *G*_3*.lock*_(*q*_3_, *q*_0_), *G*_4_(*q*_3_, *q*_4_), *G*_4*.lock*_(*q*_2_)
*L*_3_	*G*_5_(*q*_0_, *q*_1_), *G*_5*.lock*_(*q*_2_), *G*_6_(*q*_3_,*q*_2_), *G*_6*.lock*_(*q*_4_, *q*_1_)
*L*_4_	*G*_7_(*q*_1_, *q*_0_), *G*_7*.lock*_(*q*_2_)

## Data Availability

The data used to support the findings of this study will be available from the author upon request.
